# A kinetic proofreading argument to understand the role of H3K9 trimethylation in phase-separated chromatin states

**DOI:** 10.1016/j.bpj.2026.01.023

**Published:** 2026-02-09

**Authors:** Ralf Blossey, Helmut Schiessel

**Affiliations:** 1 University of Lille, Unité de Glycobiologie Structurale et Fonctionnelle (UGSF), CNRS UMR8576, Lille, France; 2 Cluster of Excellence Physics of Life, TUD Dresden University of Technology, Dresden, Germany; 3 Institut für Theoretische Physik, TUD Dresden University of Technology, Dresden, Germany

## Abstract

Histone trimethylation on the tail residue H3K9 of nucleosomes is a hallmark of constitutive heterochromatin. The mechanism of how this mark is maintained over cell division cycles remains a topic of ongoing debate. The recent formulation of the polymer-assisted condensation scenario, through which a stable droplet condensate can form as a reaction container for methylation reactions, has offered a novel approach to answer this long-standing question. In this letter, we develop a kinetic proofreading argument for histone tail methylation that builds on the polymer-assisted condensation model and yields support to the idea that liquid-liquid phase separation underlies the reconstitution of heterochromatin over cell division cycles.

## Significance

A kinetic proofreading argument is developed supporting the concept that liquid-liquid phase separation is the key ingredient in the maintenance of trimethylated H3K9 tail residues of nucleosomes in chromatin.

## Introduction

On nucleosomes, the histone tail trimethylation of the residue H3K9 is a hallmark of constitutive heterochromatin, i.e., the transcriptionally essentially silenced part of chromatin. Over the course of cell division cycles, heterochromatin needs to be passed along from the mother to the daughter cells. In this process, the nucleosomes carrying the silencing mark are distributed equally between the cells, so that in each of the daughter cells the newly synthesized nucleosomes need to be equipped with the silencing mark (1,2). But how does the enzymatic machinery of methylation target the desired substrates—that is, those nucleosomes to be found in the heterochromatin portion from those outside, typically then transcriptionally active euchromatin? The distinction between in this sense “right” and “wrong” substrates immediately makes one think of kinetic proofreading concepts, as we have employed them before for the role of histone tail modifications in transcription processes; see, e.g., (3,4,5,6). Those processes concern the action of chromatin remodelers on nucleosomes that fit naturally into the classic Hopfield scenario (7). In this original scenario, both equilibrium processes of molecular recognition and nonequilibrium aspects of ATP consumption of the remodeling motors conspire. It is the presence of the irreversible ATP consumption step that is decisive for substrate discrimination; for a review of this mechanisms, see (8). In methylation processes, ATP however plays a different role, as we explain below. The key process is a different one, which is what we argue here.

**Figure 1:**
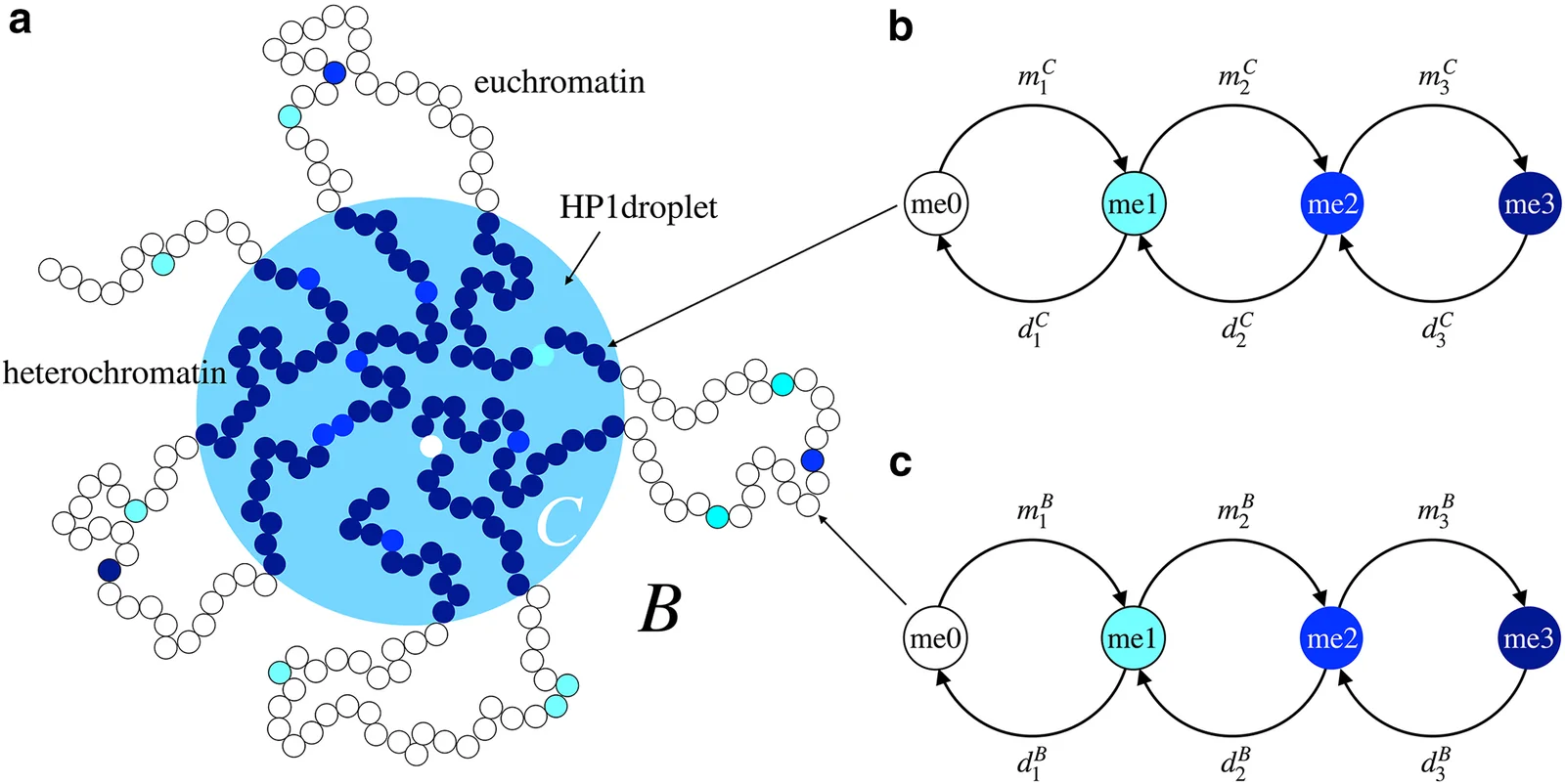
A nuclear condensate separates euchromatin and heterochromatin and controls their respective epigenetic marks. (*a*) Micellar structure of chromatin with heterochromatin blocks inducing a liquid HP1 droplet and euchromatin blocks forming loops outside. (*b*) Methylation reaction scheme for nucleosomes inside the condensate “*C*.” (*c*) Same as (*b*) but for nucleosomes in the bulk “*B*.”

Recently one of us (H.S.) has argued that the formation of biomolecular condensates through liquid-liquid phase separation, a wide-spread phenomenon in biological systems (9), plays a crucial role in structuring chromatin in a way that allows its epigenetic state to be maintained across multiple cell generations. Specifically, it is argued that chromosomes can be considered effectively as block copolymers consisting of stretches of nucleosomes with H3K9me3 marks (constitutive heterochromatin; with a median length of about 50 nucleosomes in humans (10)) and without these marks (euchromatin). Future extensions of the model are planned to account also for other types of epigenetic marks such as H3K27me3, a hallmarks of facultative heterochromatin. H3K9me3 marks have been shown to constitute binding sites for heterochromatin protein 1 (HP1) (11,12,13,14), a protein that is known to form in vitro liquid condensates at sufficient high concentrations and that also has been observed to exist as condensates inside cells (15,16). We suggested that the concentration of HP1 in vivo is so low that the system lies outside the miscibility gap; i.e., the components are in the mixed state, and no phase separation takes place spontaneously. Moreover, we argued that through the attraction of HP1 proteins to the heterochromatin stretches a condensate forms through a process called polymer-assisted condensation (PAC) (17). As a result, the chromosome forms a micellar structure with a liquid core formed from HP1 and heterochromatin stretches and a corona consisting of euchromatin loops outside this core (17,18). This leads to a situation where the two types of nucleosomes reside in different phases, the H3K9me3 nucleosomes in the HP1-rich phase and the mark-free nucleosomes in the HP1-poor phase. This situation is sketched in Fig. 1 *a*.

Cells can make use of the different chemical environments of euchromatin and heterochromatin to reestablish H3K9me3 marks that are partially lost at each DNA duplication. For this to work, cells must have a methylase with a strong preference for the H3K9me3 environment. In fact, SUV39H1, the methylase responsible for adding methyl groups to the H3K9 residue, is known to prefer HP1 (19,20). Therefore, we argued that this property allows cells to recover epigenetic histone marks that were lost during DNA duplication without adding (too many) H3K9me3 marks to euchromatin-associated nucleosomes. In a recent computational study, we actually demonstrated that this mechanism can work through 50 cell generations, the Hayflick limit, which denotes the maximal number a somatic cell can be duplicated (18).

However, in this study, we simplified the reaction scheme by assuming that the marker is set in one step and did not consider the presence of demethylation reactions. To achieve the reestablishment of the epigenetic markers, we then needed to assume a very steep dependence of the methylation rate on the HP1 concentration. In the present study, we consider the role of the methylation reactions in more detail; especially, we consider the possibility that the trimethylation mark is the result of three independent methylation reactions. Moreover, we allow demethylation reactions to compete with the methylation reactions (21). The sequence of methylation and demethylation reactions in drawn in Fig. 1 *b* and *c*. We assume that these reactions take place in two different phases and that the reaction rates are affected by these phases. We aim to answer the question of whether the discrimination ratio of the reactions to occur at the “right” and the “wrong” nucleosomes in the sense defined above is enhanced by the fact that the heterochromatic mark is only achieved with the addition of the trimethylation mark.

As already mentioned, the methylation and demethylation reactions do not have an irreversible ATP-dependent step, as is the case in chromatin remodeling. The ATP dependence of these processes is “hidden,” as it provides the energy for the synthesis of methyl donors, e.g., S-adenosylmethionine, and other cofactors (22). The kinetic proofreading scenario we propose for the phase-separated chromatin states therefore differs in this key aspect from the usual Hopfield scenario.

## Theory

In line with our introduction and the situation we envisage in Fig. 1 *a*, we consider nucleosomes that can be either in the bulk, i=B, or inside the condensate, i=C; we do not consider here the possibility of nucleosomes changing their environment; i.e., the value of i is fixed for each nucleosome. We assume that the nucleosomes can be in four different epigenetic states: nonmethylated, k=0, monomethylated, k=1, dimethylated, k=2, and trimethylated, k=3. We denote the fraction of nucleosomes in state k in phase i by $f_{k}^{i}$. One has

(1) $$f_{0}^{i}+f_{1}^{i}+f_{2}^{i}+f_{3}^{i}=1,\;\;\;i=B,C.$$

As shown in Fig. 1 *b*, we assume that nucleosomes can be methylated and demethylated one step at a time. We denote the methylation rate for a nucleosome in phase i to go from state k=j−1 to state k=j (with j=1,2,3) by $m_{j}^{i}$ and the demethylation rate to go from state k=j to state k=j−1 by $d_{j}^{i}$.

In order to develop the (classic) kinetic proofreading argument, we consider the reactions in thermodynamic equilibrium, and one has

(2) $$f_{j}^{i}=\frac{m_{j}^{i}}{d_{j}^{i}}f_{j-1}^{i}$$

for j=1,2,3. Again in the spirit of kinetic proofreading, we can now define an error ratio of “right” versus “wrong” results of the reactions in order to quantify the discrimination between the two. Here, we consider trimethylated nucleosomes inside the condensate as “right” and trimethylated nucleosomes in the bulk as “wrong.” The error ratio R is thus given by

(3) $$R=\frac{f_{3}^{C}}{f_{3}^{B}}=\left(\overset{3}{\underset{j=1}{\prod }}\frac{m_{j}^{C}}{d_{j}^{C}}\frac{d_{j}^{B}}{m_{j}^{B}}\right)\frac{f_{0}^{C}}{f_{0}^{B}}.$$

To determine $f_{0}^{C}/f_{0}^{B}$, we use Eqs. 1 and 2:

(4) $$1=f_{0}^{i}\left(1+\frac{m_{1}^{i}}{d_{1}^{i}}\left(1+\frac{m_{2}^{i}}{d_{2}^{i}}\left(1+\frac{m_{3}^{i}}{d_{3}^{i}}\right)\right)\right).$$

This finally leads to the error ratio R expressed in terms of all the methylation and demethylation rates in this system:

(5) $$R=\frac{1+\frac{m_{1}^{B}}{d_{1}^{B}}\left(1+\frac{m_{2}^{B}}{d_{2}^{B}}\left(1+\frac{m_{3}^{B}}{d_{3}^{B}}\right)\right)}{1+\frac{m_{1}^{C}}{d_{1}^{C}}\left(1+\frac{m_{2}^{C}}{d_{2}^{C}}\left(1+\frac{m_{3}^{C}}{d_{3}^{i}}\right)\right)}\overset{3}{\underset{j=1}{\prod }}\frac{m_{j}^{C}}{d_{j}^{C}}\frac{d_{j}^{B}}{m_{j}^{B}}.$$

As R depends only on the ratio of methylation and demethylation reactions, we simplify the notation by introducing $r_{j}^{i}=m_{j}^{i}/d_{j}^{i}$. In terms of these ratios, the error fraction simplifies to

(6) $$R=\frac{1+r_{1}^{B}\left(1+r_{2}^{B}\left(1+r_{3}^{B}\right)\right)}{1+r_{1}^{C}\left(1+r_{2}^{C}\left(1+r_{3}^{C}\right)\right)}\overset{3}{\underset{j=1}{\prod }}\frac{r_{j}^{C}}{r_{j}^{B}}.$$

Assuming, for simplicity, that the reaction rate ratios do not depend on the methylation step, $r_{j}^{i}=r^{{}_{i}}$ (but see Ref. (23) for the more complex biological reality), this simplifies to

(7) $$R=\frac{1+r^{B}\left(1+r^{B}\left(1+r^{B}\right)\right)}{1+r^{C}\left(1+r^{C}\left(1+r^{C}\right)\right)}{\left(\frac{r^{C}}{r^{B}}\right)}^{3}.$$

To reach a large value of R, we need according to Eq. 3 a large value of $f_{3}^{C}$ and a small value of $f_{3}^{B}$. However, $f_{3}^{C}$ cannot exceed one. The only way we can thus achieve a large R value is by having $f_{3}^{B}\ll 1$, which implies $r^{B}\ll 1$. In this case Eq. 7 simplifies to

(8) $$R≃\frac{{\left(r^{C}\right)}^{3}}{1+r^{C}\left(1+r^{C}\left(1+r^{C}\right)\right)}{\left(r^{B}\right)}^{-3}=f_{3}^{C}\times {\left(\frac{d^{B}}{m^{B}}\right)}^{3}.$$

We can estimate the parameters from the observed densities of H3K9me3 marks in heterochromatin and euchromatin. Heterochromatin typically shows a “semimarking phenomenon” of H3K9me3 marks (2), whereas these marks have a low density in euchromatin. For instance, a study on sonication resistance of chromatin suggests $f_{3}^{C}\approx 61\%$ and $f_{3}^{B}\approx 3\%$ (23,24), which leads to R≈20. This implies $r^{C}=2.46$ and $r^{B}=0.36$, which, inserted in Eq. 7, indeed recovers the R value.

## Discussion

In this letter, we have developed a kinetic proofreading argument for the role of H3K9 trimethylation. The methylation of histone residues is special compared with the other main posttranslational modification on histone tails, which is “mono”: there are, e.g., no di-acetylations. Having even a trimethylation in the histone code is therefore bound to have a specific reason. Our model for the error ratio of H3K9 trimethylation, as given by expression Eq. (8), gives a plausible rationale for its existence, as the reaction rate to assemble and disassemble the trimethylated state is translated into a power on the ratio of those rates, thus resulting in a nonlinear effect with a cubic power. It is key that this process requires the presence of two compartments generated by the previously described phase-separation process of PAC (17,18). In contrast to the Hopfield scenario as it has been applied to histone tail states, the scenario for trimethylation of H3K9 works without the explicit involvement of ATP to furnish an irreversible step: the two key ingredients here are the phase-separated states of nucleosomes in their chromatin context and the high degree of methylation, which provides a sufficiently nonlinear effect. An interesting open question remains whether there is a critical block length for H3K9me3 nucleosomes below which the mechanism described above is not viable anymore. For example, it not clear whether the mechanism can be applied also to heterochromatin nanodomains that are typically 3 to 10 nucleosomes long (25). To answer these questions will require systematic computer simulations along the lines of Ref. (18).

## Conclusion

In sum, we have demonstrated that the trimethylation of the histone tail residue H3K9 in the context of phase-separated chromatin states allows for the discrimination between “right” and “wrong” nucleosomes and hence works in favor of the maintenance of this histone mark in cell division cycles. The role of methylations of other histone tail residues, depending on their specific biological context, remains an interesting open problem in which kinetic proofreading arguments may also find their use.

## Acknowledgments

H.S. is supported by the 10.13039/501100001659Deutsche Forschungsgemeinschaft (DFG, 10.13039/501100001659German Research Foundation) under Germany’s Excellence Strategy—EXC-2068—390729961. R.B. acknowledges support from the Agence Nationale de Recherche (ANR) under grant “Dyprosome” (ANR-21-CE45-0032-02).

## Author contributions

The work was initiated during a visit by one of us (H.S.) at the Unité de Gylcobiologie Structurale et Fonctionnelle. We did it again together, very much in the spirit of (26).

## Declaration of interests

The authors declare no competing interests.

## References

[1] Cutter DiPiazza A.R., Taneja N., Grewal S.I.S. (2021). Spreading and epigenetic inheritance of heterochromatin require a critical density of histone H3 lysine 9 tri-methylation. Proc. Natl. Acad. Sci..

[2] Owen J.A., Osmanović D., Mirny L. (2023). Design principles of 3D epigenetic memory systems. Science.

[3] Blossey R., Schiessel H. (2008). Kinetic proofreading of gene activation by chromatin remodeling. HFSP J..

[4] Blossey R., Schiessel H. (2011). Kinetic Proofreading in Chromatin Remodeling: The Case of ISWI/ACF. Biophys. J..

[5] Blossey R., Schiessel H. (2019). Histone mark recognition controls nucleosome translocation via a kinetic proofreading mechanism: Confronting theory and high-throughput experiments. Phys. Rev. E.

[6] Schiessel H., Blossey R. (2020). Pioneer transcription factors in chromatin remodeling: The kinetic proofreading view. Phys. Rev. E.

[7] Hopfield J.J. (1974). Kinetic proofreading: a new mechanism for reducing errors in biosynthetic processes requiring high specificity. Proc. Natl. Acad. Sci. USA.

[8] Boeger H. (2022). Kinetic Proofreading. Annu. Rev. Biochem..

[9] Banani S.F., Lee H.O., Rosen M.K. (2017). Biomolecular condensates: organizers of cellular biochemistry. Nat. Rev. Mol. Cell Biol..

[10] Barkess G., West A.G. (2012). Chromatin insulator elements: establishing barriers to set heterochromatin boundaries. Epigenomics.

[11] Bannister A.J., Zegerman P., Kouzarides T. (2001). Selective recognition of methylated lysine 9 on histone H3 by the HP1 chromo domain. Nature.

[12] Nakayama J., Rice J.C., Grewal S.I. (2001). Role of histone H3 lysine 9 methylation in epigenetic control of heterochromatin assembly. Science.

[13] Lachner M., O'Carroll D., Jenuwein T. (2001). Methylation of histone H3 lysine 9 creates a binding site for HP1 proteins. Nature.

[14] Maeda R., Tachibana M. (2022). HP1 maintains protein stability of H3K9 methyltransferases and demethylases. EMBO Rep..

[15] Strom A.R., Emelyanov A.V., Karpen G.H. (2017). Phase separation drives heterochromatin domain formation. Nature.

[16] Larson A.G., Elnatan D., Narlikar G.J. (2017). Liquid droplet formation by HP1α suggests a role for phase separation in heterochromatin. Nature.

[17] Sommer J.-U., Merlitz H., Schiessel H. (2022). Polymer-Assisted Condensation: A Mechanism for Hetero-Chromatin Formation and Epigenetic Memory. Macromolecules.

[18] Mukherjee S., Skoruppa E., Schiessel H. (2026). A self-organised liquid reaction container for cellular memory. Adv. Sci..

[19] Scharf A.N.D., Imhof A. (2011). Every methyl counts – Epigenetic calculus. FEBS Lett..

[20] Zeng W., Ball A.R., Yokomori K. (2010). HP1: heterochromatin binding proteins working the genome. Epigenetics.

[21] Hyun K., Jeon J., Kim J. (2017). Writing, erasing and reading histone lysine methylations. Exp. Mol. Med..

[22] Teperino R., Schoonjans K., Auwerx J. (2010). Histone methyl transferases and demethylases; can they link metabolism and transcription?. Cell Metab..

[23] Nicetto D., Zaret K.S. (2019). Role of H3K9me3 heterochromatin in cell identity establishment and maintenance. Curr. Opin. Genet. Dev..

[24] Becker J.S., McCarthy R.L., Zaret K.S. (2017). Genomic and Proteomic Resolution of Heterochromatin and Its Restriction of Alternate Fate Genes. Mol. Cell.

[25] Thorn G.J., Clarkson C.T., Teif V.B. (2022). DNA sequence-dependent formation of heterochromatin nanodomains. Nat. Commun..

[26] Yanai I., Lercher M.J. (2024). It takes two to think. Nat. Biotechnol..

